# The Influence of Crosslink Density on the Failure Behavior in Amorphous Polymers by Molecular Dynamics Simulations

**DOI:** 10.3390/ma9040234

**Published:** 2016-03-25

**Authors:** Junhua Zhao, Peishi Yu, Shuhong Dong

**Affiliations:** Jiangsu Key Laboratory of Advanced Food Manufacturing Equipment and Technology, Jiangnan University, Wuxi 214122, China; psyu@jiangnan.edu.cn (P.Y.); dsh@jiangnan.edu.cn (S.D.)

**Keywords:** failure behavior, cross-linked polymers, molecular dynamics simulations

## Abstract

The crosslink density plays a key role in the mechanical response of the amorphous polymers in previous experiments. However, the mechanism of the influence is still not clear. In this paper, the influence of crosslink density on the failure behavior under tension and shear in amorphous polymers is systematically studied using molecular dynamics simulations. The present results indicate that the ultimate stresses and the broken ratios (the broken bond number to all polymer chain number ratios) increase, as well as the ultimate strains decrease with increasing crosslink density. The strain concentration is clearer with the increase of crosslink density. In other words, a higher crosslink density leads to a higher strain concentration. Hence, the higher strain concentration further reduces the fracture strain. This study implies that the mechanical properties of amorphous polymers can be dominated for different applications by altering the molecular architecture.

## 1. Introduction

Linear polymers are the most fundamental polymer molecular shapes and were extensively studied in view of their significant chemical and physical properties [1,2,3]. It is well known that the molecular architecture by networking and branching has a large effect on the dielectric, mechanical, and thermal properties of amorphous polymers [4,5]. In order to produce high-performance or multifunctional polymers, one approach is to blend chemically-different monomers, add advanced fillers, and synthesize specific molecular architectures. Recently, the polymer hydrogels can be formed by ionically and covalently cross-linked networks (note that the cross-linked molecules are also called network molecules), which have fracture energies of 9000 J/m^2^ and withstand stretches of over 20 [6]. Therefore, the mechanical properties of polymers for different applications can be provided by altering molecular architectures. For example, the strength and modulus of polymers can be raised by cross-linked molecular architectures, while their fracture toughness will be reduced [6,7,8].

To capture the main characteristics of the mechanical behavior for various cross-linked polymers, molecular dynamics (MD) simulation has been carried out to study the structural and dynamic properties of polymer processes at the atomic or molecular level, which can also be used to link these observations to their macroscopic properties [9,10,11,12]. Rottach *et al.* [13] and Yang *et al.* [14] investigated the effects of crosslink on the constitutive behavior and internal structure using united-atom (UA) MD simulations, respectively. Robbins’s group [15,16] studied the mechanical behavior of glassy polymers using coarse-grained (CG) modeling of polymer networks, in which the bead includes the lumped mass. Both experimental and UA MD results have shown that the crosslink has a great effect on the compressive response of polymer particles [17,18].

However, the tensile failure behavior of the cross-linked polymers at large deformation is still not clear from a molecular perspective in view of the limitations of above UA and CG potentials. Especially, their shear failure behavior at large deformation has been hardly studied in previous study. Furthermore, the true stress acting on the samples at such large deformation is difficult to measure in experiments. Therefore, it is a fundamental challenge to reveal the molecular origins of macroscopic fracture behavior for polymers [19,20]. The MD simulations can be performed to effectively control the testing conditions and obtain the microscopic features of polymers, which can possibly be used to design different macroscopic properties by changing the molecular architecture of polymers.

In this paper, the influence of the crosslink density on the tensile and shear failure behavior (such as broken ratio, ultimate stress and ultimate strain) in amorphous polymers is systematically studied using MD simulations. The effect of the temperature and strain rate on the failure behavior is also obtained. The mechanism of the failure behavior is revealed by the strain concentration from the mechanical response. This study indicates that the mechanical properties of amorphous polymers can be dominated for different applications by changing the molecular architecture.

## 2. Simulations Details

**Figure 1:**
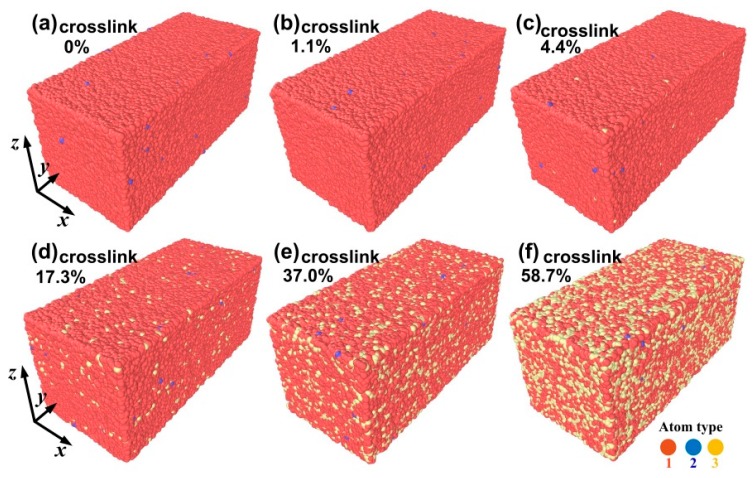
The three-dimensional molecular configuration of the crosslinked polymers consisting of 180,000 beads under uniaxial tension at *T* = 0.1 when the strain is equal to 0.8 (the three atom types of the inset represents middle bead (**red**), end bead (**blue**), and the crosslinked bead of the middle bead (**yellow**), respectively). (**a**) Configuration of linear polymers (two type beads (middle beads and end beads) can be found in the configuration); (**b**) configuration of crosslink density of 1.1%; (**c**) configuration of crosslink density of 4.4%; (**d**) configuration of crosslink density of 17.3%; (**e**) configuration of crosslink density of 37%; and (**f**) configuration of crosslink density of 58.7%.

The linear buck polymer is built by the semi-crystalline lattice method [21,22], which utilizes the face-centered cubic (fcc) diamond structure as a template to carbon backbones of entangled polymers. The modeling details can be found in previous study [22,23]. In order to avoid the effect of the thermal fluctuations around the periodic boundary layer [24], the bead number of the initial structure is kept constant at 180,000 in each sample, in which 200 linear molecular chains and 900 beads on each chain are included (note that the volume density is close to a constant and does not increase any more when the number of beads on each chain is up to 900 from previous work [25]). To obtain a representative thermoset, the chemical crosslinks are sequentially generated in the polymer melt by dispersing 1.1%, 4.4%, 17.3%, 37% and 58.7% potential crosslink sites to the beads, in which a new covalent bond is produced when the two reactive sites are found closer than a critical distance (*d*_c_ = 1.3σ) at any time [26]. The forming process of the chemical crosslink can be accelerated at increasing higher temperatures until the desired number of reactions is reached. Therefore, the samples of crosslinked polymers with different crosslink density are produced. In this study, we have studied one thermoplastic model and five thermoset models, in which the numbers of crosslinks per chain in the five thermoset models are equal to 10.1, 39.4, 155.3, 332.9 and 528.0, respectively (see Figure 1). Since it is not our purpose to study a specific polymer, a bead-spring polymer model by Kremer and Grest [27] can be adopted in this paper. A finitely-extendable nonlinear elastic (FENE) backbone potential is used among the polymer chain:

(1) $$U\left(r\right)=-\frac{k}{2}R_{0}^{2}\ln\left[1-{\left(\frac{r}{R_{0}}\right)}^{2}\right]$$

where *k* = 30 and *R*_0_ = 1.5 to ensure a certain bond stiffness and avoid chain crossing and high-frequency modes (which would require a rather small time step for the integration) [28]. The truncated Lennard–Jones (LJ) potential can be used to describe the bead interact as:

(2) $$\{\begin{array}{c} U_{\text{LJ}}\left(r\right)=4\varepsilon \left[{\left(\frac{\sigma }{r}\right)}^{12}-{\left(\frac{\sigma }{r}\right)}^{6}-{\left(\frac{\sigma }{r_{c}}\right)}^{12}+{\left(\frac{\sigma }{r_{c}}\right)}^{6}\right]\\ U_{\text{LJ}}\left(r\right)=0,r>r_{c} \end{array},r<r_{c}=1.5\sigma$$

where ε and σ are the characteristic energy and distance parameters that define the shape of the energy distance curve. *r*_c_ is the cutoff distance of the potential. At the cutoff distance, the LJ potential offers a smooth transition to zero. In this study, the simplified unit formalism is used. All physical quantities are expressed as multiples of m (bead mass), ε, σ, and *k*_b_ (Boltzmann constant), in which all these parameters are set to one in our computation [29]. The bond broken distance is set to *r* = 1.15σ and the interaction of FENE potential is shut off, and then a non-bonded LJ interaction is generated between the two beads.

Each generated initial simulation box is annealed for 1 × 10^6^ steps and controlled by the Nose-Hoover’s thermostat until the pressure and energy of the system is stable, keeping both the temperature *T* = 1.3ε/*k*_b_ and the pressure *P* = 1 (the time step *dt* = 0.002) in the NPT ensemble [30]. Afterwards, the system is cooled down to be a given temperature in NPT ensemble and the density of the system is monitored, in which an effective cooling rate 1/(1 × 10^6^ steps) should be controlled. Finally, the equilibrium box (bulk polymers) is kept at the given temperature for 1 × 10^6^ steps in the NPT ensemble. The stress-strain curves of the bulk polymers under uniaxial tension and shear at different strain rates and temperatures are obtained by performing the non-equilibrium MD simulations. The three directions are subjected to the periodic boundary conditions. LAMMPS (LAMMPS is distributed by Sandia National Laboratories, a US Department of Energy Laboratory. Details can be seen in the website http://lammps.sandia.gov/) software has been used to accomplish all MD simulations [31].

## 3. Results and Discussion

### 3.1. Failure Behavior under Uniaxial Tension

**Figure 2:**
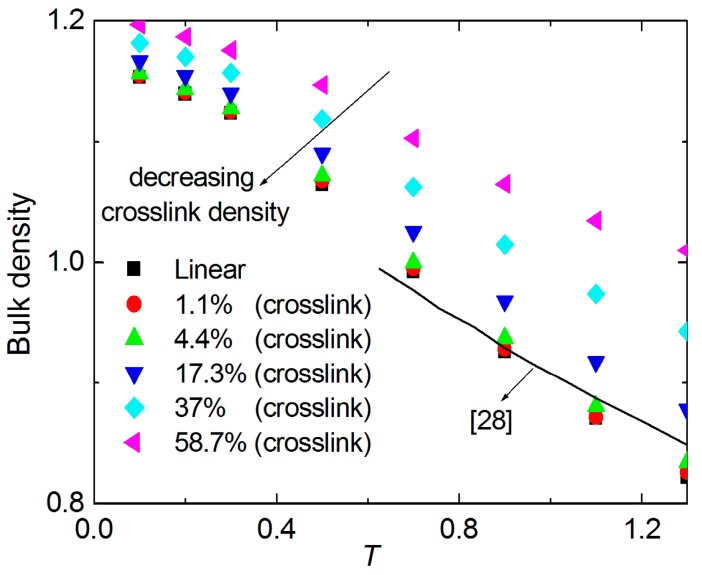
The distribution of the bulk density with various temperatures for different crosslink densities.

To validate the quality of the present structures and force field, we compare the bulk density for different crosslink densities with the available MD simulations [28] (see Figure 2). The bulk density of the linear polymer in the available study [28] is in good agreement with that of our MD simulation. The bulk density increases with the increase of crosslink density for crosslinked polymers. The higher the temperature is, the more discrete the bulk density distribution is.

**Figure 3:**
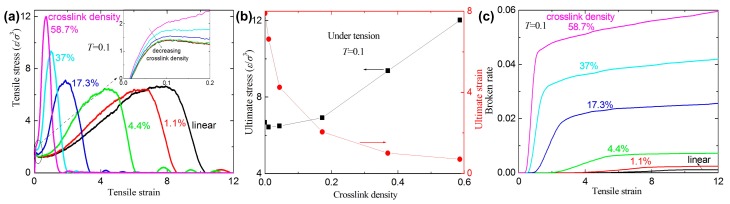
(**a**) The tensile stress-strain curves with different crosslink density at temperature *T* = 0.1; (**b**) the ultimate stress and the ultimate strain *versus* crosslink density; and (**c**) the broken ratio *versus* crosslink density at temperature *T* = 0.1.

Figure 3a shows the stress-strain curves for different crosslink density at *T* = 0.1. The slopes of the curves for the inset of Figure 3a increase with increasing crosslink density, which implies that the Young’s modulus increases with increasing crosslink density. A similar phenomenon is confirmed by previous experiments and MD simulations [17,18]. Actually, the bulk density increases with increasing crosslink density [18]. In this paper, the same size of the simulation box (the bulk cubic cell) and the same bead number of the polymers are used in the box, in which crosslink density is different. Since the lesser crosslink density leads to fewer bonds and angles in the unit cell, the demand of the total energy to reach the same strain should be smaller under uniaxial tension. The analysis further confirms that a higher crosslink density leads to a higher Young’s modulus. Figure 3b shows that the ultimate strain sharply decreases and the ultimate stress nonlinearly increases with increasing crosslink density. It indicates that higher values of the crosslink density lead to higher strength and lower fracture strain. A similar phenomenon is confirmed by previous experiments [18]. Figure 3c shows that the broken ratio increases with increasing crosslink density for a given tensile strain. The strain of the first broken bond occurred also increases with increasing crosslink density (see Figure 3c).

**Figure 4:**
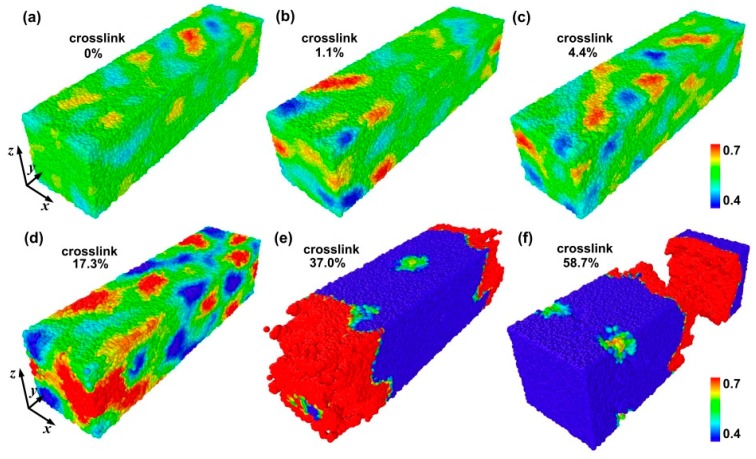
The three-dimensional atomic strain distribution for different crosslink density under tensile strain at *T* = 0.1. (**a**) Linear polymer; (**b**) 1.1% crosslink density; (**c**) 4.4% crosslink density; (**d**) 17.3% crosslink density; (**e**) 37% crosslink density; and (**f**) 58.7% crosslink density.

To further analyze the present MD simulations and understand the difference of the failure behavior for different crosslinked polymers, Figure 4 shows the three-dimensional (3D) atomic strain distribution for different crosslink density under tensile strain (the tensile strain is equal to 160%) at *T* = 0.1. The strain concentration is clearer with increasing crosslink density. In other words, a higher crosslink density leads to a higher strain concentration under the same tensile strain. Hence, the higher strain concentration further reduces the fracture strain.

**Figure 5:**
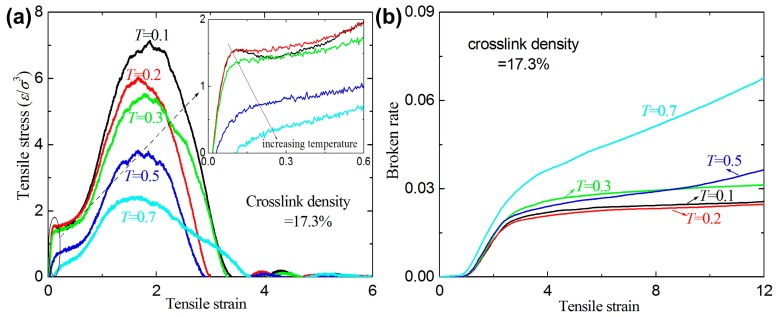
(**a**) The tensile stress-strain curves for a given crosslink density of 17.3% at different temperatures; and (**b**) the broken ratio *versus* temperature.

Figure 5 shows the stress-strain relation and broken ratio with different temperatures for a given crosslink density of 17.3%. For a given strain, the stress decreases with the increase of temperature. A similar phenomenon can be seen in linear polymers [25]. However, the broken ratio strongly depends on the temperature and the tendency with temperature is not very clear. The possible reason is that different temperature results in reorganization of the structure, which results in the entanglement density at different positions. At high temperatures of *T* = 0.5 and *T* = 0.7, the broken bond number still increases with increasing strain even at high strain (>12). Since the temperatures at *T* = 0.5 and *T* = 0.7 are both higher than the glass-transition temperature *T*_g_ = 0.35 (here), the creep and high-elastic property become more evident and the yield point disappears (see inset of Figure 5a) [21,25].

**Figure 6:**
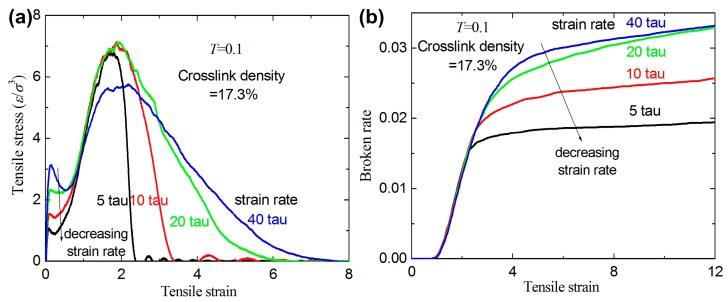
(**a**) The tensile stress-strain curves with different strain rates for a given crosslink density of 17.3%; and (**b**) the broken ratio *versus* strain rate.

Figure 6 shows the stress-strain relation and broken ratio under uniaxial tension at different strain rates for a given crosslink density 17.3%. The stress increases with increasing strain rate at small strain (0~1.5). Figure 6a shows that the yield stress at the yield point increases as the strain rate increases. A same phenomenon can be seen in linear polymers [21,25]. The broken ratios for different strain rates almost coincide when the strain is lower than 2.5, and then increases as the strain rate increases (the strain is higher than 2.5). This indicates that the broken bonds for higher strain rates are more than those for lower strain rates when the strain is higher than 2.5. Three typical mechanisms (covalent bond broken phenomenon, polymer chain slipping with each other, and separation between any two polymer chains) of the fracture behavior under uniaxial tension for amorphous polymers are provided in previous work [25]. For a smaller strain rate, the fracture process is caused by the competition between the two mechanisms of the chain slipping and broken covalent bond since more relaxation time results in more chain slipping. For higher strain rates, the fracture process is mainly dominated by the bond broken mechanism since less relaxation time results in less chain slipping. Therefore, the broken ratio for higher strain rates is higher than that for lower strain rates after the bulk polymer is completely broken.

### 3.2. Failure Behavior under Shear

**Figure 7:**
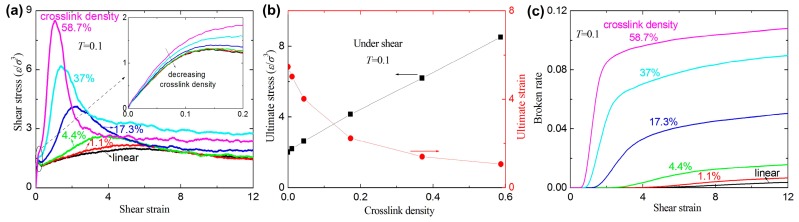
(**a**) The shear stress-strain curves with different crosslink densities at temperature *T* = 0.1; (**b**) the ultimate stress and the ultimate strain *versus* crosslink density; and (**c**) the broken ratio *versus* crosslink density at temperature *T* = 0.1.

Figure 7a shows the stress-strain curves for different crosslink density at *T* = 0.1. The slopes of the curves for the inset of Figure 7a increase with increasing crosslink density, which implies that the shear modulus increases with increasing crosslink density. A similar phenomenon is confirmed by previous experiments and MD simulations [17,18]. Actually, the bulk density increases with increasing crosslink density [18]. Since the less crosslink density leads to fewer bonds and angles in the unit cell, the demand of the total energy to reach the same strain should be also smaller under shear. The analysis also confirms that a higher crosslink density leads to a higher shear modulus. Figure 7b shows that the ultimate strain sharply decreases and the ultimate stress nonlinearly increases with increasing crosslink density. This indicates that higher values of the crosslink density lead to higher strength and lower fracture strain. A similar phenomenon is confirmed by previous experiments [17]. Figure 7c shows that the broken ratio increases with increasing crosslink density for a given shear strain. The strain of the first broken bond occurred also increases with increasing crosslink density (see Figure 7c).

**Figure 8:**
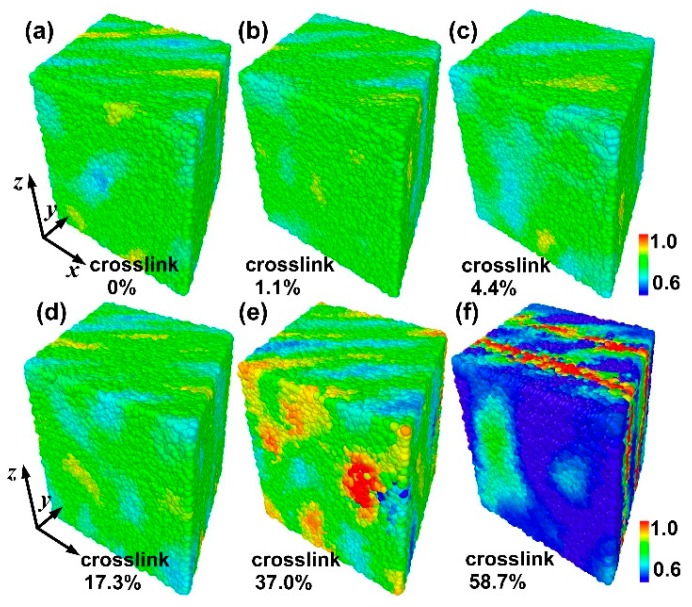
The three-dimensional atomic strain distribution for different crosslink densities under shear (=50%) at *T* = 0.1. (**a**) Linear polymer; (**b**) 1.1% crosslink density; (**c**) 4.4% crosslink density; (**d**) 17.3% crosslink density; (**e**) 37% crosslink density; and (**f**) 58.7% crosslink density.

To understand the difference of the failure behavior under shear for different crosslinked polymers, Figure 8 shows the 3D atomic strain distribution for different crosslink density under shear (shear strain is equal to 50%) at *T* = 0.1. The strain concentration is clearer with the increase of the crosslink density. In other words, a higher crosslink density leads to a higher strain concentration under the same tensile strain. Hence, the higher strain concentration further reduces the fracture strain. Moreover, the difference of the strain concentration for small crosslink density (0%, 1.1%, 4.4% and 17.3%) is not very large from Figure 8a–d. The phenomenon is further confirmed by inset of Figure 7a.

**Figure 9:**
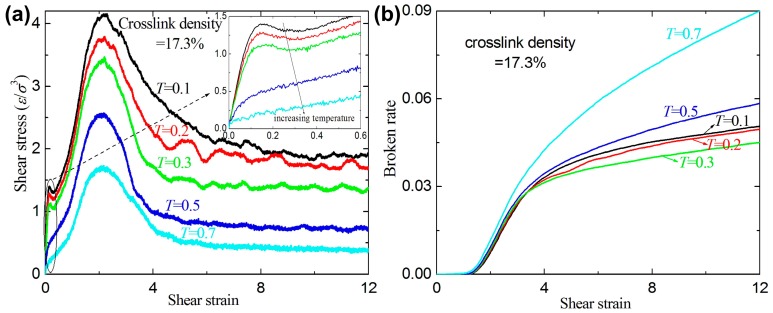
(**a**) The shear stress-strain curves for a given crosslink density of 17.3% at different temperatures; and (**b**) the broken ratio *versus* temperature.

Figure 9 shows the stress-strain relation and broken ratio with different temperatures for a given crosslink density of 17.3%. For a given strain, the stress decreases with increasing temperature. The broken ratio strongly depends on the temperature, while the rule of the temperature-dependent broken ratio is not very clear. The possible reason is that different temperatures result in reorganization of the structure, which results in the entanglement density at different positions. At the higher temperature of *T* = 0.5 and *T* = 0.7, the broken bond number also increases with increasing shear strain even at high shear strain (>12).

**Figure 10:**
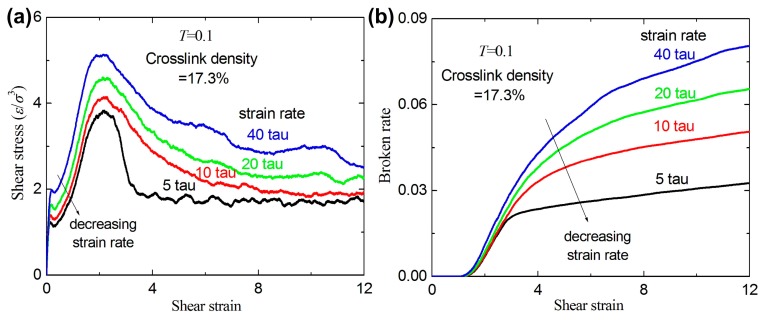
(**a**) The tensile stress-strain curves with different strain rates for a given crosslink density of 17.3%; and (**b**) the broken ratio *versus* strain rate.

Figure 10 shows the shear stress-strain relation and broken ratio for different strain rates for a given crosslink density of 17.3%. The stress increases with increasing strain rate at small strain levels (0~1.5). Figure 10a shows that the yield stress at the yield point increases as the strain rate increases. A same phenomenon can be seen in linear polymers [25]. The broken ratios for different strain rates almost coincide when the strain is lower than 2.5, and then increase as strain rates increases (the strain is higher than 2.5). This indicates that the broken bonds for higher strain rates are more than those for lower strain rates when the strain is higher than 2.5. From the above three typical mechanisms (the coupling, slipping, and broken mechanisms) of the fracture behavior [25], the strain rate effect on the fracture behavior can be similarly summarized with that under uniaxial tension. For smaller strain rates, the fracture process is controlled by the competition between the first two mechanisms. For higher strain rates, the fracture process is dominated by the first mechanism.

**Figure 11:**
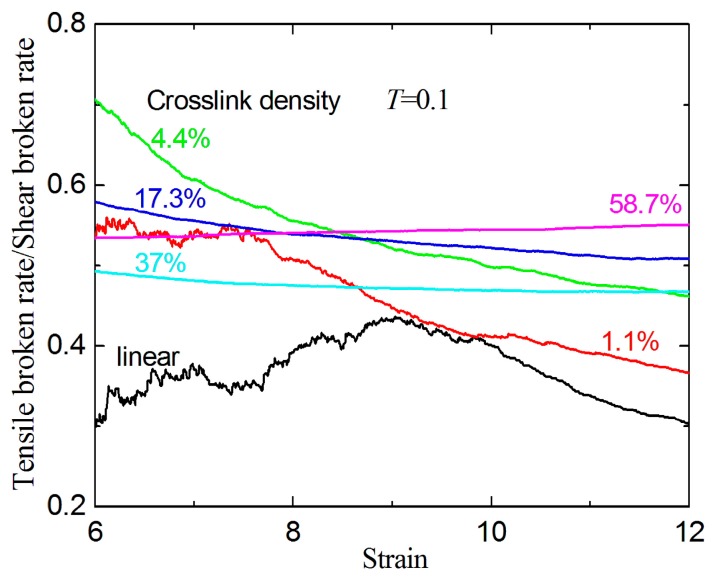
The tensile broken ratio/shear broken ratio with different crosslink densities at *T* = 0.1.

As shown in Figure 11, the ratio of broken ratio (under tension) to broken ratio (under shear) is always smaller than 1 for different strains at *T* = 0.1. For a given strain (>6) and crosslink density, the shear broken ratios are consistently higher than tensile broken ratios at the same temperature. It is possible that the first two mechanisms dominate the tensile fracture process, but the slipping and separation process causes the forward stage of the shear fracture process. Furthermore, the broken distance of *r* = 1.15σ is smaller than *r_c_* = 1.5σ of LJ potential (see Section 2), which possibly results in the higher ultimate shear fracture strain.

It should be noted that one can only obtain the qualitative mechanical properties of the crosslinked polymers using MD simulation based on the FENE potential, while the mechanism of the crosslink density effect on the mechanical properties can be effectively revealed since the parameters of the FENE potential are much less than those of the full-atom MD potentials.

## 4. Conclusions

In summary, we studied the influence of crosslink density on the tensile and shear failure behavior in amorphous polymers using MD simulations. The conclusion from the present results can be summarized as follows:

(1) The crosslink density strongly affects the mechanical response of the amorphous polymers. The ultimate stresses and the broken ratios increase with increasing crosslink density under tension and shear, while the ultimate strains decrease with increasing crosslink density.
(2) For a given crosslink density and temperature, the broken ratios and ultimate stresses increase with increasing strain rate.
(3) For a large given strain, the broken ratios under uniaxial tension are always smaller than those under shear.
